# The Interplay Between Depression, Probiotics, Diet, Immunometabolic Health, the Gut, and the Liver—A Secondary Analysis of the Pro-Demet Randomized Clinical Trial

**DOI:** 10.3390/nu16234024

**Published:** 2024-11-24

**Authors:** Oliwia Gawlik-Kotelnicka, Jakub Rogalski, Karolina H. Czarnecka-Chrebelska, Jacek Burzyński, Paulina Jakubowska, Anna Skowrońska, Dominik Strzelecki

**Affiliations:** 1 Department of Affective and Psychotic Disorders, Medical University of Lodz, 92-216 Lodz, Poland; paulina.jakubowska1@stud.umed.lodz.pl (P.J.); anna.zabka@gmail.com (A.S.); dominik.strzelecki@umed.lodz.pl (D.S.); 2 University Clinical Hospital No. 2, Medical University of Lodz, 90-549 Lodz, Poland; jakub.rogalski1@stud.umed.lodz.pl; 3 Department of Biomedicine and Genetics, Medical University of Lodz, 92-213 Lodz, Poland; karolina.czarnecka@umed.lodz.pl; 4 Department of Biostatistics and Translational Medicine, Medical University of Lodz, 92-215 Lodz, Poland; jacek.burzynski@umed.lodz.pl

**Keywords:** probiotics, liver dysfunction, cardiovascular risk, depression, intestinal permeability, diet

## Abstract

(1) Background: Depression, metabolic alternations, and liver diseases are highly comorbid. Studies have shown that probiotics might be helpful in the treatment of the above-mentioned states. The aim of this secondary analysis was to search for possible predictors of probiotics’ efficacy on liver-related outcome measures. (2) Methods: Data from 92 subjects from a randomized clinical trial on the effect of probiotics on depression were analyzed. The shift in liver steatosis and fibrosis indices was assessed in the context of baseline immunometabolic, psychometric, dietary, and intestinal permeability factors. Correlation analysis and linear regression models were used. (3) Results: A total of 30% of the variance of the improvement in the score of the aspartate transferase to platelet ratio index was explained by probiotic use, higher pre-intervention triglycerides, cholesterol, C-reactive protein levels, increased cereal intake, and a lower consumption of sweets. Then, the model of the change in alanine transferase indicated that probiotics were efficient when used by subjects with higher basal levels of intestinal permeability markers. (4) Conclusions: Probiotics being used along with a healthy diet may provide additional benefits, such as decreased cardiovascular risk, for patients with measures consistent with the immunometabolic form of depression. Probiotic augmentation may be useful for liver protection among subjects with a suspected “leaky gut” syndrome. ClinicalTrials.gov: NCT04756544.

## 1. Introduction

Depression, metabolic disorders, and numerous liver pathologies, including steatotic liver disease and drug- or alcohol-induced liver injury, often co-occur [1,2,3,4,5,6]. It is suggested that gut microbiota dysbiosis, as well as the issue of intestinal permeability (also known as “leaky gut syndrome”, a weakened gut barrier state resulting in various inflammatory agents, toxic substances, and bacterial components crossing the gut lining into systemic circulation [7]) and improper dietary habits, may mediate the clinical relationship between these disorders [8,9,10,11,12,13]. Interestingly, previous studies revealed the possible beneficial role of probiotic use and dietary interventions in treating the above-mentioned states [14,15,16,17,18]. However, there are numerous inconsistencies regarding the role of baseline metabolic and microbiota-related parameters, as well as dietary patterns, in predicting the influence of probiotics on liver-derived indices. The parental Pro-Demet randomized controlled trial (RCT), which assessed probiotics’ efficacy in depression, showed their minimal impact on depressive outcome measures as an add-on treatment dependent on the patient’s pre-treatment metabolic status, including their hepatic steatosis index (HSI) [19]. At the same time, a secondary analysis of the same RCT revealed a statistically significantly better improvement in alanine aminotransferase (ALT)-based liver biomarkers after the probiotic intervention in antidepressant-treated subjects in comparison to those not treated with antidepressive drugs. Moreover, a similar relationship was found regarding the main mental disorder diagnosis—a better improvement in fatty liver means after probiotics supplementation was observed among subjects diagnosed with depressive episodes vs. those with mixed depressive and anxiety disorder diagnoses [20].

All in all, the target subpopulation for probiotic supplementation among patients with depression is not fully understood. Specifically, it is not known whether immunometabolic features comorbid with depression, microbiota and gut permeability proxy biomarkers, or lifestyle factors are predictive of the efficacy of probiotics for liver-related non-invasive biomarkers in a clinical population with depressive disorders.

Therefore, this analysis aimed to search for possible predictors of probiotics’ influence on non-invasive biomarkers of liver steatosis and fibrosis among inflammation and metabolic parameters; intestinal permeability biomarkers—such as intestinal fatty acid-binding protein (I-FABP/FABP-2); microbiota metabolites—such as blood short-chain fatty acids (bSCFAs); or dietary habits.

We hypothesized that pre-treatment abnormalities in microbiota-intestine markers, as well as immunometabolic abnormalities comorbid with depression, would serve as possible predictors of probiotics’ efficacy for liver function based on non-invasive blood indices. Dietary habits were hypothesized to be additional explanatory factors.

## 2. Materials and Methods

### 2.1. Study Participants

A total of 116 patients with depressive disorders, according to the 11th International Classification of Diseases, were randomized and assigned to the probiotic (PRO) or placebo (PLC) groups. The PRO group received a mixture of *Lactobacillus helveticus* Rosell^®^-52 and *Bifidobacterium longum* Rosell^®^-175 for 60 days. During the trial, anthropometric, psychometric, and metabolic parameters, as well as inflammatory and microbiota proxy biomarkers, both circulating and fecal, were assessed in both groups according to the study protocol [21]. Moreover, detailed dietary habits, physical activity levels, and medication and dietary supplement intake were assessed pre-intervention.

The eligibility criteria and study timeline may be found elsewhere [21,22].

Finally, data from 92 subjects (74 women and 14 men, aged (Mdn (IQR)) 32.0 (22.5–42.1) years) were analyzed in this study. In total, 63 (68.5%) subjects were using antidepressants; the median ALT value was 16.2 (IQR: 12.9–23.3) U/L. A participant flow diagram and detailed sample characteristics of this population have been published previously [20]. A shortened form of the sample’s characteristics is shown in the Appendix A. Importantly, there were no baseline differences in their pre-intervention circulating I-FABP; bSCFAs; psychometric, metabolic, or inflammatory parameters; or dietary habits.

### 2.2. Primary Outcome Measures

Non-invasive liver-related biomarkers were analyzed (Table 1) [23,24,25].

The change in values of liver-derived blood-based biomarkers (pre-intervention vs. post-intervention) was assessed as a potential improvement/worsening in liver function in general without indicating a histopathological change in the level of steatosis/liver fibrosis [26,27].

Importantly, the aspartate aminotransferase (AST)-to-platelets ratio index (APRI) and fibrosis-4 index (FIB-4), when used in the general population, are effective in the cardiovascular risk (CVR) assessment [28,29].

The rest of the outcome measures are shown in Appendix A.

### 2.3. Statistical Analysis

Correlations between non-invasive hepatic steatosis indices and clinical parameters were assessed using Spearman’s rank correlation. We selected variables for further analysis based on Spearman’s rank correlation coefficients. To explore multivariate associations between non-invasive hepatic-related indices and other clinical parameters, we developed linear regression models with a backward feature elimination method. All independent variables were checked for collinearity and possible interactions. For each model, adjusted R^2^ was used as a measurement of explained variability. The significance threshold was set at *p* < 0.05. The significance threshold for linear regression models (six models of changes in liver-derived parameters) was set at *p* = 0.008 using Bonferroni correction.

## 3. Results

First, it was shown that variables such as age, baseline ALT, circulating lipid profile, dietary habits, physical activity, or I-FABP correlated with changes in liver steatosis and fibrosis markers values (|r| ≥ 0.20) (Table 2). Interestingly, there were noticeable differences in sets of those factors between the PRO and PLC groups.

Based on the correlation heatmap, the following factors were chosen for regression models: age, weight, diastolic blood pressure (dBP), triglycerides (TG), cholesterol, C-reactive protein (CRP), I-FABP, bSCFAs, depression subscale of Depression, Anxiety, and Stress Scale (D-DASS), fruits, oils, vegetable and seeds, sweets, meat, or cereal intake, and physical activity level.

**Figure 1:**
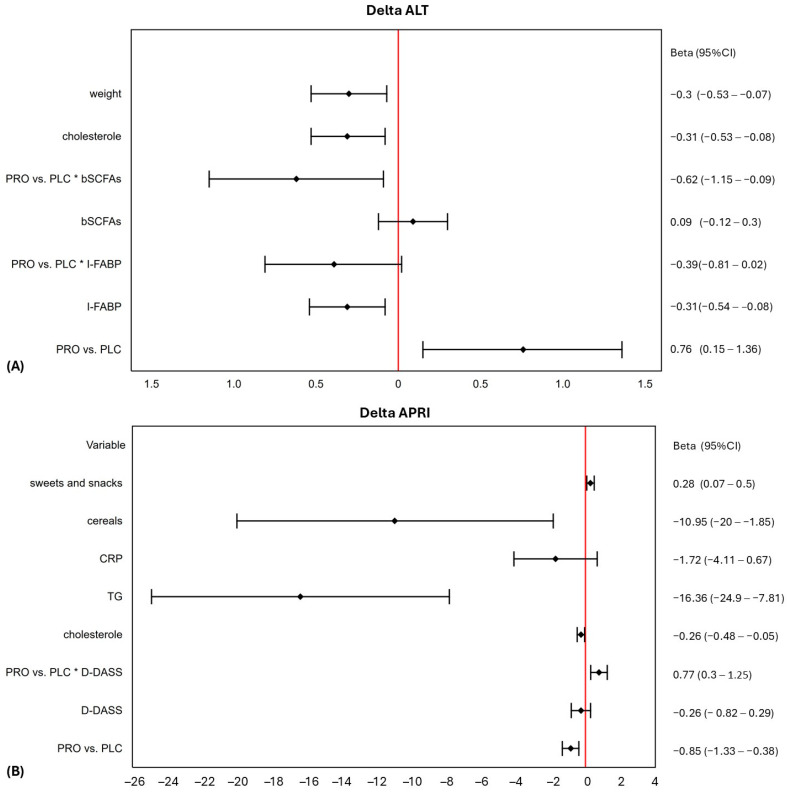
Forest plots of the multiple linear regression models with interactions, including the type of intervention (probiotic vs. placebo). (**A**) For the changes in alanine aminotransferase (ALT); (**B**) for the changes in aspartate aminotransferase (AST)-to-platelets ratio index (APRI). * means interaction. Abbreviations: bSCFAS = blood short-chain fatty acids; CRP = C-reactive protein; D-DASS = Depression subscale of Depression, Anxiety, and Stress Scale; I-FABP = intestinal fatty-acid binding protein; TG = triglycerides.

Second, the multiple linear regression (MLR) models, including PRO or PLC allocation and its interactions with other independent variables, were valid for the changes in ALT and APRI values (*p* < 0.008) (Table 3). The forest plots of MLR results are shown in Figure 1.

## 4. Discussion

### 4.1. Correlation Analyses Results

Based on the correlation analysis, as expected, pre-treatment liver steatosis and fibrosis parameters were associated with more improvement in steatosis indices values only in the PRO group.

Interestingly, the pre-intervention diet, especially fruits, vegetables, seeds, and cereal products intake, was connected with the change in liver-derived markers only in the PLC group. We hypothesize that all study participants had improved their dietary habits during the Pro-Demet intervention, as stated earlier [20]. The healthier the pre-intervention diet, the less possible any further improvement in dietary habits. Thus, participants in the PLC group gained no additional benefits from the intervention.

The higher the basal level of I-FABP, the better improvement in hepatic indices was shown in the PRO group. As FABP2 is a validated marker of enterocyte microdamage and higher intestinal permeability [30], an underlying “leaky gut” syndrome may be a proposed explanatory factor of the probiotic’s influence on liver function.

The lipid profile and CRP level were consistently shown to be connected with changes in CVR indices in the PLC, but not PRO, group, possibly making immunometabolic status not the only and not the most robust determinant of the PRO efficacy.

Finally, it is crucial to bear in mind that the above findings are only preliminary, and causal effects cannot be determined based on the results of correlation analysis. Thus, these analyses were performed to select parameters for regression models.

### 4.2. Predictors of Probiotics Possible Efficacy for Liver Dysfunction Improvement

As shown earlier, PRO intervention alone did not change ALT levels compared with PLC in the population with depressive disorders. However, significant differences in probiotics efficacy for this parameter were found when stratified subjects by the antidepressant use [20]. Here, our MLR model for the change in ALT values indicated that PRO acted differently when used by subjects with higher basal levels of I-FABP or bSCFAs, and these compounds were proposed to be indicators of the microbiota–gut wall homeostasis. I-FABP is utilized as a marker of the enterocyte lesion and the “leaky gut” syndrome [30,31]. On the other hand, circulating SCFAs, intestinal microbiota metabolites, may provide information on the gut ecosystem status—their levels and ratios are proposed to be biomarkers of the chronic inflammatory diseases state or treatment efficacy [32,33,34]. Thus, our study’s probiotic intervention effect may depend on the basal microbiota–intestinal function. Indeed, it has been demonstrated repeatedly that probiotics improve the pre-intervention gut microbiota composition and intestinal barrier status, especially following disruptive events [35,36]. Moreover, it was shown that restoring the gut microbiota balance with rifaximin helped to reduce the rate of increased intestinal permeability and decrease liver-derived inflammation and liver-related outcomes in animal models [37]. Therefore, we conclude that probiotics may act similarly on the microbiota–gut–liver axis in humans. Moreover, excessive inflammatory cytokines release, mediated by the hepato-cytotoxic injury [38], might constitute a target for probiotics’ anti-inflammatory properties, as probiotics were shown to decrease not only ALT values but also CRP levels in non-alcoholic fatty liver disease (NAFLD) subjects [39]. A recent systematic review has pointed to the curative action of probiotics for endocrine disorders through modulating inflammation [40]. Further, our previous analysis had shown that in the PRO group, subjects treated with antidepressants had greater improvement in ALT values than those who did not receive antidepressants [20]. Moreover, we have previously found increased I-FABP values under the antidepressant treatment in the Pro-Demet entry population [41]. Indeed, antidepressants were shown to lower microbiota richness and diversity as well as increase intestinal permeability in pre-clinical models [42,43,44]. All of the above-mentioned findings make the current analysis results complementary to previous ones.

To sum up, probiotics might be beneficial for liver tests in patients with depression comorbid with microbiota–gut dysfunction. Nevertheless, the adjusted R^2^ in the MLR model was found to be relatively small, indicating that other factors, not included in our study, may also have a significant impact on ALT changes.

### 4.3. The Assessment of Probiotics Efficacy and Diet in the Cardiovascular Risk Reduction

As for CVR indices, 30% of the variance of the improvement in APRI score may be explained by higher pre-intervention TG, cholesterol and CRP levels, high cereals intake and low sweets consumption, and probiotics use. Higher levels of depressive symptoms, however, counteracted the curative action of probiotics. These findings have confirmed some of the previous studies results that among people with immunometabolic alternations, probiotics in combination with other nutrients or the improvement of specific lifestyle changes, especially dietary habits, may result in better outcomes [45,46,47]. For instance, the consumption of a whole-grain pasta lowered plasma CRP, or the low-to-high-density lipoprotein cholesterol ratio (LDL/HDL) in obese volunteers, compared to pasta without a synbiotic [46]. Indeed, whole-grain cereal products contain dietary fiber, which acts as a prebiotic, and dietary fiber was found to be associated with a decreased risk of all-cause mortality [48,49]. Thus, the supplementation of probiotics along with a diet rich in fiber may act similarly to the intake of synbiotics. Regarding sweets intake, a diet low in added fructose was shown to have favorable effects on NAFLD patients [18]. Sweets are usually fortified not only with different sugars but also with artificial sweeteners, colors or preservatives. Both added sugars and artificial additives are well recognized as some of the factors increasing cardiovascular risk [50,51]. So, they may counteract the benefits of probiotics. As regards lifestyle modification in a broad sense, nutraceutical supplementation including probiotics decreased a 10-year CVR score by 40% compared to lifestyle changes alone [47]. Concerning whole dietary approaches, a dietary weight loss program, combined with probiotics supplementation, had favorable effects on total cholesterol levels in patients with coronary artery disease [52]. Moreover, our analysis results revealed that the probiotic intervention might be effective when used by participants with low baseline depressiveness. This may be because more severe depression was previously shown to be connected with poor dietary habits, e.g., a higher intake of sweets and fast food, as well as low compliance with the Mediterranean diet [53] or higher consumption of sugar-sweetened beverages in adolescents [54]. Specifically, anhedonia or anxiety was found to drive the high consumption of fried or sugar-enriched food products [55]. Thus, dietary and possibly other lifestyle improvements combined with probiotics may be necessary to affect clinical outcomes, especially in people with immunometabolic abnormalities. This may be due to fiber-derived microbiota metabolites, mainly SCFAs, which have been shown to possess anti-inflammatory properties, regulating metabolic health and even protecting the liver [56,57,58].

So, healthy dietary habits in patients with depression and immunometabolic disturbances may condition the improvement of CVR after probiotics.

### 4.4. The Generalizability of the Results

The generalizability of the findings to other populations may be, however, limited. First, the study population consisted of individuals with mild to moderate depressive disorders, the majority of whom used antidepressants. Many studies have shown that both depression and its pharmacological treatment are associated with distinct gut microbial profiles and altered gut wall permeability [42,59,60]. Similarly, metabolic abnormalities, low-grade inflammation, and unhealthy diet are more common among patients with depression than in the general population [55,61]. Second, the study population was primarily female, and chronic liver dysfunction is more prevalent in males than females due to several potential factors, including sex hormones or alcohol abuse [62].

Moreover, the long-term effects of probiotic intervention for liver function remain uncertain. In the systematic review, half of the patients with dysbiosis at enrollment were shown to have improved gut microbiota composition after the probiotic supplementation. Still, the follow-up period lasted only up to 30 days [36]. This would resemble the situation of those patients in our study with higher levels of FABP2 or bSCFAs that showed some improvement in ALT values. As this improvement is hypothesized to be associated with the decreased severity of dysbiosis and ‘leaky gut’, as discussed earlier, an ALT improvement might last as long as the gut microbiota balance. Longer-term studies in adult populations are lacking [63]. However, a probiotic effect may last long when administered very early in life; e.g., perinatal probiotic supplementation resulted in a very long-term (up to several years) decreased prevalence of allergy in children [64]. But the results of different studies are conflicting [65].

### 4.5. Limitations

The main limitation of our study is its modest sample size; thus, some analyses, especially when examining subgroups or interactions, might be underpowered. This fact limits making firm conclusions or assessing causal effects. Additionally, the explanatory nature of this secondary analysis of the primary trial data makes conclusions about the cause-and-effect relationship even more constricted. Furthermore, we used only non-invasive blood tests to assess liver function, hepatic steatosis, or any other specific liver dysfunction. Moreover, probiotic effects are strain-specific; psychobiotic strains were used due to the design of the parent study [66]. However, a meta-analysis focusing on the pro- or synbiotics influence on liver enzymes revealed that there was a massive diversity of probiotic strains used among NAFLD patients, the most common genera were *Lactobacillus*, *Bifidobacterium*, and *Streptococcus*; thus, the overlap with psychobiotics seems to be significant [65]. Nonetheless, the results of our study may enable the design of better future trial protocols to elucidate predictors of probiotics efficacy for liver-related markers in clinical populations with depression.

Despite the above-listed limitations, the strength of the present study lies in its novelty in terms of analyzed biomarkers and preliminary assessment of the complex net of interaction between clinical depression, liver, gut wall, microbiota, and diet. To the best of our knowledge, this is the first study that assessed possible conditions for probiotics action toward proxy liver abnormalities markers in patients with depressive disorders.

## 5. Conclusions

As regards patients with depression, PRO augmentation may be useful for liver protection among subjects with the suspected “leaky gut” syndrome. Further, the PRO intervention combined with the fiber-rich diet may provide additional benefits, such as a decrease in CVR, among patients with immunometabolic alterations.

The interplay between probiotics, diet, microbiota, gut, depression, and liver function may constitute the direction of future research.

## Figures and Tables

**Table 1:** Non-invasive blood-based liver function biomarkers.

The Name of the Non-Invasive Liver-Related Biomarker	Abbreviation	Liver Abnormality Detection	Formula	Cut-Off Point for Detecting/Ruling Out Liver Abnormalities
Alanine Transaminase	ALT	(acute/chronic) hepatic cytotoxic injury	-	the upper limit of normal
Aspartate Aminotransferase	AST		-	the upper limit of normal
Alanine Aminotransferase to Aspartate Aminotransferase Ratio	ALT/AST ratio	fatty liver disease	[ALT value]/[AST value]	1.33
Hepatic Steatosis Index	HSI		8 × [ALT value]/[AST value] + [BMI in kg/m^2^] + 2 * + 2 **	36
Framingham Steatosis Index	FSI		7.981 + 0.011 × [age] − 0.146 × [se×] *** + 0.173 × [BMI in kg/m^2^] + 0.007 × [TG in mg/dL] + 0.593 × [HTN] **** + 0.789 × [DM] ***** + 1.1 × [ALT/AST ratio] ******	−1.2
AST to Platelet Ratio Index	APRI	liver fibrosis	[AST value]/[AST value upper limit of normal]/[PLT value] × 100	0.5
Fibrosis-4 Index	FIB-4		[age] × [AST value]/[PLT value] × √[ALT value]	1.3

Abbreviations: ALT = alanine transaminase; APRI = AST-to-platelet ratio index; AST = aspartate aminotransferase; BMI = body mass index; DM = diabetes mellitus, FIB-4 = fibrosis-4 index; FSI = Framingham Steatosis Index; HSI = hepatic steatosis index; HTN = hypertension; PLT = platelets count; TG = triglycerides. * if DM diagnosis; ** if female; *** if female = 1, if male = 0; **** if HTN = 1, if no HTN = 0; ***** if DM = 1, if no DM = 0; ****** if ALT/AST ratio ≥ 1.33 = yes, if no = 0.

**Table 2:** Spearman’s correlation between changes in liver-derived markers and baseline factors; * *p* < 0.05, ** *p* < 0.01, *** *p* < 0.001; red—the higher value of a baseline parameter is a possible positive predictor of the decrease in liver-related marker level; green—the higher value of the parameter is a possible negative predictor of the decrease in liver-related marker level. *n* = 92.

R	≥0.40	0.30 to 0.39	0.20 to 0.29	0.10 to 0.19	−0.09 to 0.09	−0.10 to −0.19	−0.20 to −0.29	−0.30 to −0.39	≤−0.40
PRO									
			% ΔHSI	% ΔALT	% ΔALT/AST	% ΔFSI	% ΔAPRI	% ΔFIB-4	
HSI			−0.04	−0.06	−0.06	0.04	−0.06	0.02	
ALT			−0.33 *	−0.33 *	−0.34 *	0.06	−0.17	0.12	
ALT/AST			−0.25	−0.22	−0.26	0.01	−0.03	0.16	
FSI			0.07	−0.03	0.04	0.18	−0.12	−0.09	
APRI			−0.38 **	−0.45 **	−0.45 **	−0.07	−0.26	0.17	
FIB-4			−0.35 *	−0.25	−0.35 *	0.13	0.05	0.27	
AST			−0.41 **	−0.56 ***	−0.46 ***	0.05	−0.35 *	0.1	
age			−0.37 **	−0.15	−0.28	0.21	0.14	0.30 *	
sBP			0.16	0.13	0.15	0.09	0.01	−0.2	
dBP			0.27	0.21	0.23	0.01	0.1	−0.21	
weight			0.22	0.06	0.2	0.04	−0.16	−0.19	
VAI			0.2	0.1	0.22	0.25	−0.17	−0.33 *	
fGlc			−0.22	−0.15	−0.17	−0.02	0.03	0.27	
TG			0.15	0.05	0.15	0.35 *	−0.19	−0.33 *	
HDL-c			−0.2	−0.1	−0.22	0.11	0.04	0.17	
LDL-c			0.05	0.08	0.1	0.17	0.21	0.07	
Non-HDL-c			0.06	0.06	0.11	0.16	0.14	−0.04	
CHOL			−0.01	0	0.03	0.19	0.13	0.01	
TG/HDL-c			0.17	0.08	0.2	0.32 *	−0.17	−0.34 *	
TyG			0.05	−0.01	0.06	0.30 *	−0.2	−0.22	
CRP			−0.09	−0.14	−0.11	−0.09	−0.17	−0.04	
I-FABP			−0.28 *	−0.43 **	−0.39 **	0.09	−0.32 *	0.01	
bSCFAs			−0.05	−0.1	−0.11	0.04	0.16	0.18	
fruits			0.07	0.08	−0.01	0.02	0.25	0.13	
vegetables and seeds			0.09	−0.03	0.01	0.03	−0.04	0.05	
oils			0.04	−0.01	0.06	0.05	−0.05	−0.04	
sweets			0.08	0.14	0.02	−0.04	0.27	0.18	
meat			−0.04	−0.15	−0.06	−0.02	−0.05	−0.01	
diary and eggs			−0.08	0.08	−0.05	−0.02	0.11	0.14	
cereal			0.19	0.14	0.23	−0.14	−0.1	−0.22	
drinks			0.2	0.09	0.11	−0.18	0.04	−0.05	
processed food products			0.17	0.09	0.11	−0.16	0.07	−0.02	
physical activity			0.02	0.05	−0.03	−0.32	−0.14	0.07	
MADRS			0.06	0.12	0.03	0.14	0.22	0.1	
D-DASS			0.17	0.22	0.06	−0.1	0.27	0.11	
S-DASS			−0.03	−0.07	−0.11	0.08	0.05	0.22	
PLC									
			% ΔHSI	% ΔALT	% ΔALT/AST	% ΔFSI	% ΔAPRI	% ΔFIB-4	
HSI			−0.19	−0.22	−0.23	−0.15	−0.18	0.01	
ALT			−0.14	−0.31 *	−0.24	−0.1	−0.3	−0.09	
ALT/AST			−0.23	−0.36 *	−0.33 *	−0.04	−0.1	0.11	
FSI			−0.07	−0.19	−0.09	−0.05	−0.29	−0.13	
APRI			−0.12	−0.18	−0.19	−0.25	−0.33*	−0.19	
FIB-4			0.06	0.02	−0.05	−0.11	−0.16	−0.13	
AST			−0.07	−0.26	−0.13	−0.12	−0.42 **	−0.24	
age			−0.04	−0.08	−0.12	0.1	−0.09	−0.01	
sBP			0.12	−0.1	0.02	−0.17	−0.25	−0.2	
dBP			0.05	−0.11	0.1	−0.01	−0.23	−0.17	
weight			−0.08	−0.13	−0.1	−0.16	−0.26	−0.1	
VAI			0.13	0.01	0.15	0.02	−0.26	−0.22	
fGlc			−0.02	0.07	−0.01	−0.35 *	−0.06	−0.12	
TG			0.11	−0.06	0.18	0.16	−0.33 *	−0.28	
HDL-c			−0.09	−0.14	−0.13	0.18	−0.1	0.01	
LDL-c			0.2	−0.18	0.13	0.14	−0.45 **	−0.3	
non-HDL-c			0.2	−0.15	0.17	0.12	−0.46 **	−0.35 *	
CHOL			0.17	−0.2	0.11	0.15	−0.49 **	−0.32 *	
TG/HDL-c			0.17	0.03	0.23	0.09	−0.2	−0.21	
TyG			0.14	−0.01	0.2	0.1	−0.26	−0.25	
CRP			0.05	−0.13	−0.01	0.03	−0.33 *	−0.26	
I-FABP			−0.16	−0.15	−0.23	0.07	0.07	0.21	
bSCFAs			0.23	0.37 *	0.27	0.21	0.08	0	
fruits			0.09	0.2	0.05	−0.17	0.46 **	0.39 *	
oils			−0.01	0.19	−0.03	0.07	0.34 *	0.2	
vegetables and seeds			0.3	0.34 *	0.26	−0.2	0.33 *	0.15	
sweets			−0.08	−0.12	−0.09	0.27	0.09	−0.06	
meat			0.17	0.25	0.21	0.09	0.01	−0.19	
diary and eggs			0.13	0.08	−0.01	0.04	−0.12	−0.28	
cereal			0.34 *	0.35 *	0.32 *	−0.1	0.1	−0.08	
drinks			0.04	0.28	0.23	0.01	0.02	−0.15	
processed food products			0.11	0.16	0.18	0.19	−0.01	−0.25	
physical activity			−0.38	−0.28	−0.47	0.3	0	0.51 *	
(*n* = 41)									
MADRS			0.02	0.16	0.06	−0.12	0.11	0.03	
D-DASS			−0.06	−0.03	0.03	−0.02	−0.34 *	−0.3	
S-DASS			−0.1	0.02	−0.14	0.12	−0.05	0.1	

Abbreviations: ALT—alanine aminotransferase; APRI—AST-to-platelet ratio; AST—aspartate aminotransferase; bSCFAs—blood short-chain fatty acids; CHOL—cholesterol; CRP—C-reactive protein; dBP—diastolic blood pressure; D-DASS—Depression subscale of the Depression, Anxiety, and Stress Scale; fGlc—fasting serum glucose; FIB-4—Fibrosis-4 Index; FSI—Framingham Steatosis Index; HDL-c—high-density lipoprotein cholesterol; HSI—Hepatic Steatosis Index; I-FABP—intestinal fatty acid-binding protein; LDL-c—low-density lipoprotein cholesterol; MADRS—Montgomery Asberg Depression Rating Scale; PLC—placebo group; PRO—probiotic group; sBP—systolic blood pressure; S-DASS—Stress subscale of the Depression, Anxiety, and Stress Scale; TG—triglycerides; TyG—triglyceride–glucose index; VAI—visceral adiposity index.

**Table 3:** The summary of the multiple linear regression models with interactions for the change in liver steatosis or cardiovascular risk-related indices, including the type of intervention (probiotic vs. placebo).

Dependent Variable	R^2^adj	F	*p*	Independent Variable	Coefficient (β [95% CI])	*p*-Value
Δ ALT	0.186	3.74	0.001	PRO vs. PLC	0.76 (0.15–1.36)	0.014
				I-FABP	−0.31 (−0.54–−0.08)	0.008
				PRO vs. PLC * I-FABP	−0.39 (−0.81–0.02)	0.061
				bSCFAs	0.09 (−0.12–0.30)	0.378
				PRO vs. PLC * bSCFAs	−0.62 (−1.15–−0.09)	0.023
				cholesterol	−0.31 (−0.53–−0.08)	0.009
				weight	−0.30 (−0.53–−0.07)	0.010
Δ APRI	0.304	4.88	<0.001	PRO vs. PLC	−0.85 (−1.33–−0.38)	<0.001
				D-DASS	−0.26 (−0.82–0.29)	0.349
				PRO vs. PLC * D-DASS	0.77 (0.30–1.25)	0.002
				cholesterol	−0.26 (−0.48–−0.05)	0.018
				TG	−16.36 (−24.91–−7.81)	<0.001
				CRP	−1.72 (−4.11–0.67)	0.157
				cereals	−10.95 (−20.06–−1.85)	0.019
				sweets and snacks	0.28 (0.07–0.50)	0.010

Symbols: Δ—the change between the post- and pre-intervention values. Abbreviations: bSCFAs—blood short-chain fatty acids; CRP—C-reactive protein; D-DASS—Depression subscale of the Depression, Anxiety, and Stress Scale; HDL-c—high-density lipoprotein cholesterol; I-FABP—intestinal fatty acid-binding protein; PLC—placebo group; PRO—probiotic group; R^2^adj—adjusted squared coefficient of determination; TG—triglycerides; VAI—visceral adiposity index. * means interaction.

## Data Availability

The dataset is available from the corresponding author upon request.

## Appendix A

### Appendix A.1

**1. Sample Characteristics (*n* = 92) Parameter**	**Number (%) or Mdn (IQR)**
MetS according to IDF	20 (21.74%)
Antidepressants	63 (68.47%)
Antipsychotics	4 (4.35%)
Other pharmacological treatment	33 (35.87%)
Comorbidities	48 (52.17%)
Smoking status	13 (4.13%)
Dietary supplements	48 (52.17%)
Physical activity (MET-min/week)	1783.50 (1026.50–2892.00)
ALT (U/L)	16.20 (12.90–23.30)
ALT/AST	0.75 (0.63–0.89)
HSI	32.13 (28.49–35.14)
FSI	−2.64 (−3.50–−1.61)
APRI	0.24 (0.19–0.30)
FIB-4	0.61 (0.46–0.80)
Weight (kg)	68.30 (56.50–79.60)
BMI (kg/m^2^)	24.31 (21.21–27.15)
WC (cm)	83.25 (72.00–93.50)
fGlc (mmol/L)	5.07 (4.86–5.38)
HDL-c (mmol/L)	1.61 (1.43–1.90)
non-HDL-c (mmol/L)	3.60 (2.88–4.36)
TG (mmol/L)	0.95 (0.80–1.39)
Sweets and snacks	2.57 (2.14–3.14)
Diary and eggs	3.17 (2.67–3.67)
Cereal products	3.20 (2.60–3.40)
Oils	2.67 (2.33–3.00)
Fruits	2.70 (2.40–3.20)
Vegetables and seeds	3.36 (2.91–3.82)
Meat (including fish)	2.25 (1.86–2.75)
Drinks (excluding water)	2.00 (1.71–2.43)
Processed food products	2.36 (2.10–2.71)
MADRS score	19.00 (16.00–24.00)
DASS score	64.50 (47.00–80.00)
Depression	21.00 (14.00–27.00)
Anxiety	16.00 (10.00–23.00)
Stress	24.00 (20.00–34.00)
CRP (mg/L)	1.20 (0.50–2.85)
TNF-α (pg/mL)	6.00 (1.84–7.57)
I-FABP (ng/mL)	1843.38 (1169.14–2485.55)
bSCFAs (pg/mL)	4341.41 (3376.19–5820.68)
Dietary habits: 1—never or almost never; 2—once a month; 3—several times a month; 4—several times a week; 5—every day; 6—several times a day. Abbreviations: ALT = alanine aminotransferase; APRI = aspartate aminotransferase-to-platelets ratio index; BMI = body mass index; bSCFAs = blood short-chain fatty acids; CRP = C-reactive protein; DASS = Depression, Anxiety, and Stress Scale; fGlc = fasting glucose; FSI = Framingham Steatosis Index; HDL-c = HDL cholesterol; HSI = hepatic steatosis index; IDF = International Diabetes Federation; I-FABP = intestinal fatty acid-binding protein; IQR = interquartile range; MADRS = Montgomery–Asberg Depression Rating Scale; Mdn = median; MET = metabolic equivalent of task; MetS = metabolic syndrome; TG = triglycerides; TNF-α = tumor necrosis factor alpha; WC = waist circumference.	

### Appendix A.2. Secondary Outcomes

Delta (Δ) was defined as a post-(V2) minus pre-intervention (V1) value.

%delta (%Δ) was defined as (Δ/V1) * 100%.

The Depression, Anxiety, and Stress Scale (DASS) was used to measure three negative emotional states. This analysis used data measured by the Depression subscale of DASS (D-DASS) [67].

The triglycerides to high-density lipoprotein cholesterol (TG/HDL-c) ratio has been proposed as a marker of insulin resistance and MetS occurrence [68].

The visceral adiposity index (VAI) is a sex-specific index including WC, BMI, TG and HDL-c to estimate visceral adiposity. It is calculated by using the following formulas: for males: VAI = WC [cm]/(39.68 + (1.88 × BMI) × (TG [mmol/L]/1.03) × (1.31/HDL-c [mmol/L]); for females: VAI = WC [cm]/(36.58 + (1.89 × BMI)) × (TG [mmol/L]/0.81) × (1.52/HDL-c [mmol/L]) [69].

TyG index is a proxy marker for identifying insulin resistance. It is calculated as Ln(fasting TG [mg/dL]  × fasting blood glucose [mg/dL]/2 [70].

Intestinal fatty acid-binding protein (FABP2/I-FABP) increased levels occur when an intestinal epithelial cell lesion is found; it is used as a marker of “leaky gut” [71].

Short-chain fatty acids (SCFAs) include butyrate, propionate, and acetate, and are metabolites of gut microbiota produced from fibers. They play a role in supporting the intestinal barrier, preventing neuroinflammation and metabolic dysfunctions [72].

## References

[1] Sahoo S., Mishra E., Premkumar M. (2024). Antidepressants in People with Chronic Liver Disease and Depression: When Are They Warranted and How to Choose the Suitable One?. J. Clin. Exp. Hepatol..

[2] Ueberberg B., Frommberger U., Messer T., Zwanzger P., Kuhn J., Anghelescu I., Ackermann K., Assion H.J. (2020). Drug-Induced Liver Injury (DILI) in Patients with Depression Treated with Antidepressants: A Retrospective Multicenter Study. Pharmacopsychiatry.

[3] Hsu J.H., Chien I.C., Lin C.H. (2019). Increased Risk of Chronic Liver Disease in Patients with Major Depressive Disorder: A Population-Based Study. J. Affect. Disord..

[4] Gu Y., Zhang W., Hu Y., Chen Y., Shi J. (2022). Association between Nonalcoholic Fatty Liver Disease and Depression: A Systematic Review and Meta-Analysis of Observational Studies. J. Affect. Disord..

[5] Huang X., Liu X., Yu Y. (2017). Depression and Chronic Liver Diseases: Are There Shared Underlying Mechanisms?. Front. Mol. Neurosci..

[6] Manikat R., Nguyen M.H. (2023). Nonalcoholic Fatty Liver Disease and Non-Liver Comorbidities. Clin. Mol. Hepatol..

[7] Dmytriv T.R., Storey K.B., Lushchak V.I. (2024). Intestinal Barrier Permeability: The Influence of Gut Microbiota, Nutrition, and Exercise. Front. Physiol..

[8] Kronsten V.T., Tranah T.H., Pariante C., Shawcross D.L. (2022). Gut-Derived Systemic Inflammation as a Driver of Depression in Chronic Liver Disease. J. Hepatol..

[9] Liu L., Wang H., Chen X., Zhang Y., Zhang H., Xie P. (2023). Gut Microbiota and Its Metabolites in Depression: From Pathogenesis to Treatment. eBioMedicine.

[10] Schwenger K.J., Clermont-Dejean N., Allard J.P. (2019). The Role of the Gut Microbiome in Chronic Liver Disease: The Clinical Evidence Revised. JHEP Rep..

[11] Fang J., Yu C.H., Li X.J., Yao J.M., Fang Z.Y., Yoon S.H., Yu W.Y. (2022). Gut Dysbiosis in Nonalcoholic Fatty Liver Disease: Pathogenesis, Diagnosis, and Therapeutic Implications. Front. Cell Infect. Microbiol..

[12] Mega A., Marzi L., Kob M., Piccin A., Floreani A. (2021). Food and Nutrition in the Pathogenesis of Liver Damage. Nutrients.

[13] Mrozek W., Socha J., Sidorowicz K., Skrok A., Syrytczyk A., Piątkowska-Chmiel I., Herbet M. (2023). Pathogenesis and Treatment of Depression: Role of Diet in Prevention and Therapy. Nutrition.

[14] Bischoff S.C., Bernal W., Dasarathy S., Merli M., Plank L.D., Schütz T., Plauth M. (2020). ESPEN Practical Guideline: Clinical Nutrition in Liver Disease. Clin. Nutr..

[15] Firth J., Marx W., Dash S., Carney R., Teasdale S.B., Solmi M., Stubbs B., Schuch F.B., Carvalho A.F., Jacka F. (2019). The Effects of Dietary Improvement on Symptoms of Depression and Anxiety: A Meta-Analysis of Randomized Controlled Trials. Psychosom. Med..

[16] Merkouris E., Mavroudi T., Miliotas D., Tsiptsios D., Serdari A., Christidi F., Doskas T.K., Mueller C., Tsamakis K. (2024). Probiotics’ Effects in the Treatment of Anxiety and Depression: A Comprehensive Review of 2014–2023 Clinical Trials. Microorganisms.

[17] Maslennikov R., Ivashkin V., Efremova I., Poluektova E., Shirokova E. (2021). Probiotics in Hepatology: An Update. World J. Hepatol..

[18] Semmler G., Datz C., Trauner M. (2023). Eating, Diet, and Nutrition for the Treatment of Non-Alcoholic Fatty Liver Disease. Clin. Mol. Hepatol..

[19] Gawlik-Kotelnicka O., Margulska A., Płeska K., Skowró Nska A., Strzelecki D. (2024). Metabolic Status Influences Probiotic Efficacy for Depression—PRO-DEMET Randomized Clinical Trial Results. Nutrients.

[20] Gawlik-Kotelnicka O., Burzyński J., Rogalski J., Skowrońska A., Strzelecki D. (2024). Probiotics May Be Useful for Drug-Induced Liver Dysfunction in Patients with Depression–A Secondary Analysis of a Randomized Clinical Trial. Clin. Nutr. ESPEN.

[21] Gawlik-Kotelnicka O., Skowrońska A., Margulska A., Czarnecka-Chrebelska K.H., Łoniewski I., Skonieczna-Żydecka K., Strzelecki D. (2021). The Influence of Probiotic Supplementation on Depressive Symptoms, Inflammation, and Oxidative Stress Parameters and Fecal Microbiota in Patients with Depression Depending on Metabolic Syndrome Comorbidity—PRO-DEMET Randomized Study Protocol. J. Clin. Med..

[22] Gawlik-Kotelnicka O., Margulska A., Skowrońska A., Strzelecki D. (2023). PRO-DEMET Randomized Controlled Trial on Probiotics in Depression—Pilot Study Results. Nutrients.

[23] Long M.T., Pedley A., Colantonio L.D., Massaro J.M., Hoffmann U., Muntner P., Fox C.S. (2016). Development and Validation of the Framingham Steatosis Index to Identify Persons with Hepatic Steatosis. Clin. Gastroenterol. Hepatol..

[24] Lee J.H., Kim D., Kim H.J., Lee C.H., Yang J.I., Kim W., Kim Y.J., Yoon J.H., Cho S.H., Sung M.W. (2010). Hepatic Steatosis Index: A Simple Screening Tool Reflecting Nonalcoholic Fatty Liver Disease. Dig. Liver Dis..

[25] Park J.H., Choi J., Jun D.W., Han S.W., Yeo Y.H., Nguyen M.H. (2019). Low Alanine Aminotransferase Cut-Off for Predicting Liver Outcomes; A Nationwide Population-Based Longitudinal Cohort Study. J. Clin. Med..

[26] Basheer M., Naffaa M., Assy N. (2023). Non-Invasive Biomarkers of Liver Fibrosis in Nonalcoholic Fatty Liver Disease. Clin. Mol. Hepatol..

[27] Jang H., Kim Y., Lee D.H., Joo S.K., Koo B.K., Lim S., Lee W., Kim W. (2024). Outcomes of Various Classes of Oral Antidiabetic Drugs on Nonalcoholic Fatty Liver Disease. JAMA Intern. Med..

[28] Schreiner A.D., Zhang J., Moran W.P., Koch D.G., Marsden J., Livingston S., Mauldin P.D., Gebregziabher M. (2023). FIB-4 and Incident Severe Liver Outcomes in Patients with Undiagnosed Chronic Liver Disease: A Fine-Gray Competing Risk Analysis. Liver Int..

[29] De Matteis C., Cariello M., Graziano G., Battaglia S., Suppressa P., Piazzolla G., Sabbà C., Moschetta A. (2021). AST to Platelet Ratio Index (APRI) Is an Easy-to-Use Predictor Score for Cardiovascular Risk in Metabolic Subjects. Sci. Rep..

[30] Arnone D. (2023). Increased Levels of Intestinal-Type Fatty Acid-Binding Protein (I-FABP) in Mood Disorders. Psychol. Med..

[31] Lau E., Marques C., Pestana D., Santoalha M., Carvalho D., Freitas P., Calhau C. (2016). The Role of I-FABP as a Biomarker of Intestinal Barrier Dysfunction Driven by Gut Microbiota Changes in Obesity. Nutr. Metab..

[32] Saresella M., Marventano I., Barone M., La Rosa F., Piancone F., Mendozzi L., d’Arma A., Rossi V., Pugnetti L., Roda G. (2020). Alterations in Circulating Fatty Acid Are Associated with Gut Microbiota Dysbiosis and Inflammation in Multiple Sclerosis. Front. Immunol..

[33] Deleu S., Machiels K., Raes J., Verbeke K., Vermeire S. (2021). Short Chain Fatty Acids and Its Producing Organisms: An Overlooked Therapy for IBD?. eBioMedicine.

[34] Gandhi P., Gandhi I., Hoeschen A., Mosher W., MacMillan M.L., Rashidi A., El Jurdi N.H., Khoruts A., Weisdorf D.J., Blazar B.R. (2020). Plasma Short Chain Fatty Acids as a Predictor of Response to Therapy for Life-Threatening Acute Graft-Versus-Host Disease. Blood.

[35] Zheng Y., Zhang Z., Tang P., Wu Y., Zhang A., Li D., Wang C.Z., Wan J.Y., Yao H., Yuan C.S. (2023). Probiotics Fortify Intestinal Barrier Function: A Systematic Review and Meta-Analysis of Randomized Trials. Front. Immunol..

[36] McFarland L.V. (2014). Use of Probiotics to Correct Dysbiosis of Normal Microbiota Following Disease or Disruptive Events: A Systematic Review. BMJ Open.

[37] Han J.W., Kim D.I., Nam H.C., Chang U.I., Yang J.M., Song D.S. (2021). Association between Serum Tumor Necrosis Factor-α and Sarcopenia in Liver Cirrhosis. Clin. Mol. Hepatol..

[38] Yan M., Man S., Ma L., Guo L., Huang L., Gao W. (2024). Immunological Mechanisms in Steatotic Liver Diseases: An Overview and Clinical Perspectives. Clin. Mol. Hepatol..

[39] Huang Y., Wang X., Zhang L., Zheng K., Xiong J., Li J., Cong C., Gong Z., Mao J. (2022). Effect of Probiotics Therapy on Nonalcoholic Fatty Liver Disease. Comput. Math. Methods Med..

[40] Nemati M., Ebrahimi B., Montazeri-Najafabady N. (2024). Probiotics Ameliorate Endocrine Disorders via Modulating Inflammatory Pathways: A Systematic Review. Genes. Nutr..

[41] Gawlik-Kotelnicka O., Czarnecka-Chrebelska K., Margulska A., Pikus E., Wasiak J., Skowrońska A., Brzeziańska-Lasota E., Strzelecki D. (2025). Associations between Intestinal Fatty-Acid Binding Protein and Clinical and Metabolic Characteristics of Depression. Prog. Neuropsychopharmacol. Biol. Psychiatry.

[42] Cussotto S., Strain C.R., Fouhy F., Strain R.G., Peterson V.L., Clarke G., Stanton C., Dinan T.G., Cryan J.F. (2019). Differential Effects of Psychotropic Drugs on Microbiome Composition and Gastrointestinal Function. Psychopharmacology.

[43] Hatamnejad M.R., Baradaran Ghavami S., Shirvani M., Asghari Ahmadabad M., Shahrokh S., Farmani M., Sherkat G., Asadzadeh Aghdaei H., Zali M.R. (2022). Selective Serotonin Reuptake Inhibitors and Inflammatory Bowel Disease; Beneficial or Malpractice. Front. Immunol..

[44] Sjöstedt P., Enander J., Isung J. (2021). Serotonin Reuptake Inhibitors and the Gut Microbiome: Significance of the Gut Microbiome in Relation to Mechanism of Action, Treatment Response, Side Effects, and Tachyphylaxis. Front. Psychiatry.

[45] Dang L., Li D., Mu Q., Zhang N., Li C., Wang M., Tian H., Jha R., Li C. (2024). Youth-Derived Lactobacillus Rhamnosus with Prebiotic Xylo-Oligosaccharide Exhibits Anti-Hyperlipidemic Effects as a Novel Synbiotic. Food Res. Int..

[46] Angelino D., Martina A., Rosi A., Veronesi L., Antonini M., Mennella I., Vitaglione P., Grioni S., Brighenti F., Zavaroni I. (2019). Glucose- and Lipid-Related Biomarkers Are Affected in Healthy Obese or Hyperglycemic Adults Consuming a Whole-Grain Pasta Enriched in Prebiotics and Probiotics: A 12-Week Randomized Controlled Trial. J. Nutr..

[47] Dahlberg C.J., Ou J.J., Babish J.G., Lamb J.J., Eliason S., Brabazon H., Gao W., Kaadige M.R., Tripp M.L. (2017). A 13-Week Low Glycemic Load Diet and Lifestyle Modification Program Combining Low Glycemic Load Protein Shakes and Targeted Nutraceuticals Improved Weight Loss and Cardio-Metabolic Risk Factors. Can. J. Physiol. Pharmacol..

[48] Garzón A.G., Veras F.F., Brandelli A., Drago S.R. (2024). Bio-Functional and Prebiotics Properties of Products Based on Whole Grain Sorghum Fermented with Lactic Acid Bacteria. J. Sci. Food Agric..

[49] Ramezani F., Pourghazi F., Eslami M., Gholami M., Mohammadian Khonsari N., Ejtahed H.S., Larijani B., Qorbani M. (2024). Dietary Fiber Intake and All-Cause and Cause-Specific Mortality: An Updated Systematic Review and Meta-Analysis of Prospective Cohort Studies. Clin. Nutr..

[50] Girigosavi K.B., Etta I., Kambham S., Panjiyar B.K. (2024). Sweet Surprises: An In-Depth Systematic Review of Artificial Sweeteners and Their Association with Cerebrovascular Accidents. Curr. Nutr. Rep..

[51] Yeterian M., Parikh M.A., Frishman W.H., Peterson S.J. (2024). The Bittersweet Reality: The Cardiovascular Risk of Artificial Sweeteners. Cardiol. Rev..

[52] Moludi J., Alizadeh M., Behrooz M., Maleki V., Seyed Mohammadzad M.H., Golmohammadi A. (2021). Interactive Effect of Probiotics Supplementation and Weight Loss Diet on Metabolic Syndrome Features in Patients with Coronary Artery Diseases: A Double-Blind, Placebo-Controlled, Randomized Clinical Trial. Am. J. Lifestyle Med..

[53] Paans N.P.G., Gibson-Smith D., Bot M., van Strien T., Brouwer I.A., Visser M., Penninx B.W.J.H. (2019). Depression and Eating Styles Are Independently Associated with Dietary Intake. Appetite.

[54] Dabravolskaj J., Patte K.A., Yamamoto S., Leatherdale S.T., Veugelers P.J., Maximova K. (2024). Association Between Diet and Mental Health Outcomes in a Sample of 13,887 Adolescents in Canada. Prev. Chronic Dis..

[55] Alrehaili S., Afifi A.A., Algheshairy R.M., Bushnaq T., Alharbi T.A.F., Alharbi H.F. (2024). Prevalence of Anhedonia, Anxiety, and Their Impact on Food Consumption among Postgraduate Qassim University Students. Front. Nutr..

[56] Olaniyi K.S., Areloegbe S.E., Badejogbin O.C., Ajadi I.O., Ajadi M.B. (2024). Butyrate-Mediated Modulation of Paraoxonase-1 Alleviates Cardiorenometabolic Abnormalities in a Rat Model of Polycystic Ovarian Syndrome. Cardiovasc. Drugs Ther..

[57] Ziętek M., Celewicz Z., Kikut J., Szczuko M. (2021). Implications of Scfas on the Parameters of the Lipid and Hepatic Profile in Pregnant Women. Nutrients.

[58] Tan J.K., Macia L., Mackay C.R. (2023). Dietary Fiber and SCFAs in the Regulation of Mucosal Immunity. J. Allergy Clin. Immunol..

[59] Wasiak J., Gawlik-Kotelnicka O. (2023). Intestinal Permeability and Its Significance in Psychiatric Disorders–A Narrative Review and Future Perspectives. Behav. Brain Res..

[60] Gamboa J., Le G.H., Wong S., Alteza E.A., Zachos K.A., Teopiz K.M., McIntyre R.S. (2024). Impact of Antidepressants on the Composition of the Gut Microbiome: A Systematic Review and Meta-Analysis of in Vivo Studies. J. Affect. Disord..

[61] Teixeira A.L., Scholl J.N., Bauer M.E. (2024). Psychoneuroimmunology of Mood Disorders. Methods Mol. Biol..

[62] Cooper K.M., Delk M., Devuni D., Sarkar M. (2023). Sex Differences in Chronic Liver Disease and Benign Liver Lesions. JHEP Rep..

[63] Merenstein D., Pot B., Leyer G., Ouwehand A.C., Preidis G.A., Elkins C.A., Hill C., Lewis Z.T., Shane A.L., Zmora N. (2023). Emerging Issues in Probiotic Safety: 2023 Perspectives. Gut Microbes.

[64] Lundelin K., Poussa T., Salminen S., Isolauri E. (2017). Long-Term Safety and Efficacy of Perinatal Probiotic Intervention: Evidence from a Follow-up Study of Four Randomized, Double-Blind, Placebo-Controlled Trials. Pediatr. Allergy Immunol..

[65] Davies G., Jordan S., Brooks C.J., Thayer D., Storey M., Morgan G., Allen S., Garaiova I., Plummer S., Gravenor M. (2018). Long Term Extension of a Randomised Controlled Trial of Probiotics Using Electronic Health Records. Sci. Rep..

[66] Darwesh M.A.K., Bakr W., Omar T.E.I., El-Kholy M.A., Azzam N.F. (2024). Unraveling the Relative Abundance of Psychobiotic Bacteria in Children with Autism Spectrum Disorder. Sci. Rep..

[67] Makara-Studzińska M., Załuski M., Amczyk K.A.D., Tyburski E. (2024). Polish Version of the Depression Anxiety Stress Scale (DASS-42)-Adaptation and Normalization. Psychiatr. Pol..

[68] Kosmas C.E., Rodriguez Polanco S., Bousvarou M.D., Papakonstantinou E.J., Peña Genao E., Guzman E., Kostara C.E. (2023). The Triglyceride/High-Density Lipoprotein Cholesterol (TG/HDL-C) Ratio as a Risk Marker for Metabolic Syndrome and Cardiovascular Disease. Diagnostics.

[69] Amato M.C., Giordano C., Galia M., Criscimanna A., Vitabile S., Midiri M., Galluzzo A. (2010). Visceral Adiposity Index: A Reliable Indicator of Visceral Fat Function Associated with Cardiometabolic Risk. Diabetes Care.

[70] Simental-Mendía L.E., Rodríguez-Morán M., Guerrero-Romero F. (2008). The Product of Fasting Glucose and Triglycerides as Surrogate for Identifying Insulin Resistance in Apparently Healthy Subjects. Metab. Syndr. Relat. Disord..

[71] Stevens B.R., Goel R., Seungbum K., Richards E.M., Holbert R.C., Pepine C.J., Raizada M.K. (2018). Increased Human Intestinal Barrier Permeability Plasma Biomarkers Zonulin and FABP2 Correlated with Plasma LPS and Altered Gut Microbiome in Anxiety or Depression. Gut.

[72] Yu S., Wang L., Jing X., Wang Y., An C. (2023). Features of Gut Microbiota and Short-Chain Fatty Acids in Patients with First-Episode Depression and Their Relationship with the Clinical Symptoms. Front. Psychol..

